# School-based gatekeeper training programmes in enhancing gatekeepers’ cognitions and behaviours for adolescent suicide prevention: a systematic review

**DOI:** 10.1186/s13034-018-0233-4

**Published:** 2018-06-07

**Authors:** Phoenix K. H. Mo, Ting Ting Ko, Mei Qi Xin

**Affiliations:** 1 Division of Behavioral Health and Health Promotion, School of Public Health and Primary Care, Faculty of Medicine, The Chinese University of Hong Kong, Shatin, N. T., Hong Kong; 2 Faculty of Medicine, School of Public Health and Primary Care, The Chinese University of Hong Kong, Shatin, N. T., Hong Kong

**Keywords:** Adolescents, Gatekeeper training, School-based, Suicide prevention, Systematic review

## Abstract

Suicide is a leading cause of death in adolescence. School provides an effective avenue both for reaching adolescents and for gatekeeper training. This enables gatekeepers to recognize and respond to at-risk students and is a meaningful focus for the provision of suicide prevention. This study provides the first systematic review on the effectiveness of school-based gatekeeper training in enhancing gatekeeper-related outcomes. A total of 815 studies were identified through four databases (Ovid Medline, Embase, PsycINFO and ERIC) using three groups of keywords: ‘school based’, ‘Suicide prevention programme’ and ‘Gatekeeper’. Fourteen of these studies were found to be adequate for inclusion in this systematic review. The improvement in gatekeepers’ knowledge; attitudes; self-efficacy; skills; and likelihood to intervene were found in most of the included studies. Evidence of achieving improvement in attitudes and gatekeeper behaviour was mixed. Most included studies were methodologically weak. Gatekeeper training appears to have the potential to change participants’ knowledge and skills in suicide prevention, but more studies of better quality are needed to determine its effectiveness in changing gatekeepers’ attitudes. There is also an urgent need to investigate how best improvements in knowledge and skills can be translated into behavioural change.

## Background

### Adolescent suicide as a significant public health issue

Suicide-related behaviour is common among school-aged adolescents. Globally, suicide is reported to be the second leading cause of death among young people aged 15–29 [1]. It is believed that the suicide rate is underreported in many countries due to inconsistent death classification systems, and the cultural and religious beliefs that may affect the coroner’s decisions [2, 3].

### Associated factors and consequences of adolescent suicide

Adolescent suicide is a serious and complex public health problem which is associated with a range of interlocking factors. Facing the shift to middle school or high school, students have to adapt to a new environment in many aspects [4]. However, some adolescents are not mature enough to deal with this kind of life transition, leading to substance or alcohol abuse [4, 5], depression, unruly behaviour such as bullying and fighting or even expulsions by their schools [6]. These are all risk factors for suicidal behaviour. Also, conflicts with family members, relationship problems with close friends, and uncertainty about the future are identified as trigger points for suicidal behaviour [7]. The impact of losing a young life not only causes huge societal loss but also brings tremendous psychological suffering to their families [8]. Suicide may even create a copycat effect due to the sensational reporting by media, especially in Asia [9]. Interventions to prevent adolescent suicide-related behaviour are highly warranted.

### Importance of school-based intervention in preventing adolescent suicide

Reducing adolescent suicide is a huge challenge in many countries. Many adolescents who have suicidal thoughts are not willing to seek help [10, 11]. They also avoid attending the treatment arranged for them [12], and are less likely to seek help from formal channels [13]. Although many suicide prevention programmes are available in the community, it is often difficult to reach those suicidal youths to provide resources and support. In view of these challenges, school-based programmes are recommended for adolescents as they can provide an easy on-going access to students [14]. As adolescents spend most of their time in school, school-based programmes are considered one of the most effective ways to address the problem of adolescent suicide and to promote help-seeking among adolescents [15].

Most school-based suicide prevention programmes fall into one of three categories. First, suicide awareness education curricula aims to increase students’ awareness of suicide, help students recognize the signs of suicide, and encourage self-disclosure [16]. One criticism of this approach, however, is that increasing students’ knowledge and awareness of suicide does not necessarily lead to behavioural change [17]. Second, peer leadership training programmes train students to help their suicidal peers by responding appropriately and referring them to a trusted adult [18]. However, a peer leader may not be able to approach their suicidal peers as those who have suicidal thoughts usually isolate themselves from the peer network, limiting the efficacy of the programme [18]. Third, screening programmes can help to identify at-risk students for suicide prevention [17]. A valid and reliable screening tool is important to prevent the potential iatrogenic effect. Review on suicide prevention programmes reported that limited evidence exists in suggesting that education and screening is effective in reducing suicide [19]. Furthermore, for those suicide prevention programmes that are found to be effective, most of them have their effects diminished over time.

### Gatekeeper approach as a promising way for adolescent suicide prevention

More recently, the gatekeeper approach has been recognized as a promising way for adolescent suicide prevention. Gatekeepers are defined as “individuals in a community who have face-to-face contact with large numbers of community members as part of their usual routine”. The gatekeeper approach therefore aims to train those gatekeepers to identify individuals who are at-risk of suicide and refer them to health care professionals [20]. Gatekeeper training programmes are developed as many individuals who have suicidal ideation do not seek help, and that risk factors for suicide are recognizable and thus identifiable [18]. In a school setting, gatekeeper training is a widely disseminated strategy that trains gatekeepers to recognize signs of suicide, and enhances knowledge and attitudes to intervene with at-risk students [13]. Through the gatekeeper training programme, participants have the ability to respond appropriately and effectively to those at-risk students, so that early identification and referral to health professionals can be achieved [21]. Furthermore, gatekeeper training relies on outside service and stakeholders’ support, such as mental health services and treatment [22].

Some suicide prevention programmes are created under the gatekeeper training principles, for example, in the primary gatekeeper training programme, Question, Persuade, Refer (QPR) [23], participants learn the suicidal warning signs, as well as the skills to assess at-risk students, to manage the situation appropriately and to refer them to health professionals for treatment if necessary. Although it has been identified as the best practice, a rigorous evaluation on this approach remains scarce [17]. Another prominent gatekeeper training programme, Applied Suicide Intervention Skills Training (ASIST), is a 2 day interaction workshop for participants to gradually build comfort and understanding about suicide and suicide intervention [24].

Main participants of gatekeeper programme are school personnel, such as teachers, teaching staff, coaches and administrators. There is no doubt that adolescents spend most of their time in school every day. School personnel also play an important role on youth growth and have lots of opportunities to contact and interact with students. They can observe any abnormal behavior from students and offer them support. On the other hand, it has also been shown that most of the teachers feel uncomfortable and unprepared about addressing the topic of suicide. They report a lack of skills to respond when coping with students’ suicidal signs and behaviour [25, 26]. The gatekeeper approach is therefore a potentially effective method to increase their knowledge and skills in dealing with adolescents who are at-risk of suicide [27].

The gatekeeper approach is frequently used in attempts to reduce rates of adolescent suicide. The extent to which it is effective in achieving this, especially in a school-based setting, remains unclear [28]. Although there is evidence that gatekeeper training can improve the knowledge and attitudes of participants [29] and is recommended in school-based suicide prevention, some studies failed to demonstrate the effectiveness of this programme [30]. Increase in knowledge and attitude may not enable the school staff to effectively recognize and respond to some students’ suicidality without explicit warning signs. It was further argued that students with suicidal ideation are less likely to seek help through school personnel compared with other students, thus universal gatekeeper training that merely focused on the staff’s roles may not be sufficient for the success of suicide prevention [29]. A review to synthesize the evidence of school-based gatekeeper training for adolescent suicide prevention is warranted.

## Aims

‘Despite its implementation in many settings, a systematic evaluation on the efficacy of this approach in adolescent suicide prevention is currently lacking [31]. With the different content and methods used in various studies, a systematic review can synthesize the findings and provide clear evidence on whether school-based gatekeeper training is an effective method of suicide prevention among adolescents. The current study aims to conduct a systematic review on the effectiveness of school-based gatekeeper training in enhancing gatekeepers’ knowledge, skills, attitudes, and behaviour for adolescent suicide prevention.

## Methods

### Identification of relevant studies

Studies related to school-based gatekeeper training for adolescent suicide prevention were identified from four online databases, namely Ovid Medline (1946–2017 December 18), Embase (1910–2017 December 18), PsycINFO (1806–2017 December Week 2) and ERIC (1966–2017 December 19). The search was restricted to English articles and studies of all types, including journal articles, book chapters, and dissertations were included. Bibliographies of the included studies and a systematic review on gatekeeper training for suicide prevention [32] were also examined for further relevant studies.

A broad search strategy was employed and search keywords were categorized into three key terms: “school-based”, “suicide prevention programme”, and “gatekeeper”. To maximize the search in the databases, various synonyms and combinations of the search terms were used. Search terms for “school-based” included “school”, or “curriculum based”. Search terms for “Suicide prevention programme” included “suicide prevention”, “suicide education”, “self-harm prevention”, or “suicide intervention”. Search terms for “gatekeeper” included “gatekeeper”, “teacher”, “staff”, “personnel”, “counsellor”, “psychologist”, “Question, Persuade, Refer”, or “Applied Suicide Intervention Skills Training”.

### Inclusion and exclusion criteria

Studies were included for the review if they: (1) used a controlled trial (RCT) or quasi-experiment design; (2) primarily targeted suicide prevention; (3) used a gatekeeper approach for the intervention, in which more than 60% of the participants of the programme are school personnel who have face to face contact with students; (4) were based in middle school or high school; (5) had at least one outcome related to suicide prevention (see below section for details); and, (6) contained a comparison group or reported pre- and post-intervention data. No restrictions on the eligibility of studies were imposed on the basis of sample size, duration of follow-up, or publication source.

### Exclusion criteria

Studies were excluded if they were: (1) non-school based; (2) not related to suicide prevention; (3) general suicide prevention programmes without using a gatekeeper approach; (4) using peer as gatekeeper; (5) non-intervention based (e.g. qualitative studies, commentary, or review); (6) using a single group design with only post-intervention data reported; or (7) not written in English.

### Study outcome

Various outcomes for suicide prevention training have been identified in the literature. Due to the low frequency of completed suicide and the difficulty in ascertaining suicide rate [25], reducing suicide rate should not be regarded as the key indicator for effectiveness of a suicide prevention programme [33]. In the context of gatekeeper training programmes for suicide prevention, the most common outcomes included increase in gatekeepers’ knowledge of suicide risk assessment and management, improvement in skills of observing any abnormal signs and dealing with at-risk individuals appropriately [34], increase in confidence in dealing with individuals who are at risk of suicide, and positive gains in attitude towards suicide. Gatekeeper behaviors related to intervening with suicidal individuals, such as speaking with students who are at risk of suicide, or referring students to mental health services, were also measured [35].

Based on the current literature of suicide prevention using a gatekeeper approach, the following gatekeeper-related outcomes were included in the review: knowledge about adolescent suicide, gatekeeper skills, attitudes towards adolescent suicide, self-efficacy, likelihood to intervene when a student has suicidal thoughts, and gatekeeper behaviours.

### Data extraction

Two reviewers independently reviewed and screened the articles. Disagreements were resolved by discussion. Data were extracted using a coding scheme designed by the authors and the following information was coded: location of the study, sample characteristics, intervention characteristics, measures used, and outcomes. Effect size (Cohen’s d) was directly extracted or computed by using the raw data for each test [36]. For studies with a design of ‘controlled trial without a pre-test’ or ‘before- and after comparison’, Cohen’s d was estimated as the mean difference divided by the pooled standard deviation (SD), with an adjustment to unequal sample size as appropriate [37]. For studies with a design of ‘controlled trial with pre- and post-test’, the estimation was based on the pooled pre-test SD across intervention conditions [38]. If means and SDs were not available, other indices of effect size were extracted and converted to Cohen’s d (e.g. t, partial eta-squared) [36, 39]. An assessment of the quality of studies with comparison groups was also conducted. This included their use of randomized assignment, concealment methods, use of an intent-to-treat analysis, and whether the intervention deliverer was blinded to the study.

## Results

### Included studies

**Fig. 1:**
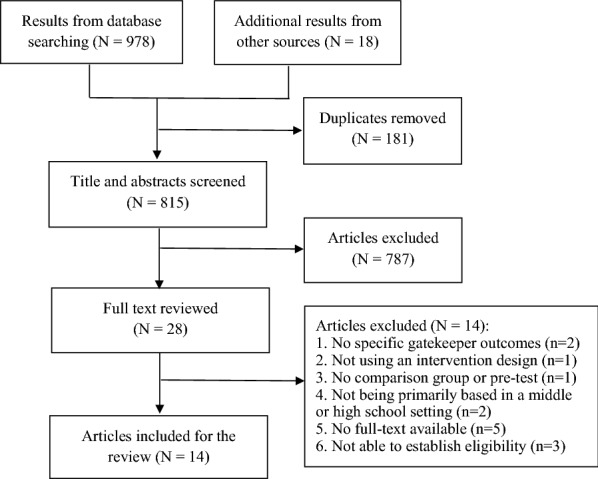
Flow chart of screening process

The database search identified 978 studies with a further 18 found through screening the bibliographies of the relevant literature; 181 of these were duplicate and thus removed. The titles and abstracts of the remaining 815 studies were screened; 28 of these were relevant to the study aims and retained for examination of the full text. Despite efforts to contact the authors for full text or more study details, these could not be obtained for five from any available source, and adequate information to establish study eligibility could not be obtained for three others. Finally, 14 studies met all inclusion criteria and were included in the review (Fig. 1; Table 1).

**Table 1:** Characteristics and main results of the studies included in the systematic review (N = 14)

	Participants					Intervention							
Study	Location	Sample size	Sample type	Mean age	% of male	Name of the programme	Intervention group (INT)	Comparison group (COM)	Program duration and attrition rate at post-test^3^	Follow-up duration and attrition rate at follow-up	Outcomes	Instruments	Main results
*Controlled trials with pre- and post-test*													
Cross et al. [13]	New York, United States	INT = 72 CON = 75	School staff (N = 91) and parents (N = 56)	School staff = 42.07 (SD = 10.41) Parents = 43.49 (SD = 4.65)	School staff = 23.1% Parents = 5.4%	QPR	Gatekeeper training plus behavioral rehearsal	QPR	INT = 1 h 25 min; NA CON = 1 h; NA	3 months	1. Knowledge	Declarative knowledge: Adapted from previous studies [52, 53]; 14 items Self-perceived knowledge: Adapted from previous studies [52, 54, 55]; 5 items	Significant increase in both groups at post-test (d = 0.61 for INT; d = 0.74 for COM) and maintained at follow-up (d = 0.57 for INT; d = 0.46 for COM); no group (d = − 0.11 at post-test; d = 0.12 at follow-up) or interaction effects were found Significant increase in both groups at post-test (d = 2.08 for INT; d = 2.01 for COM) and maintained at follow-up (d = 1.86 for INT; d = 1.63 for COM); no group (d = 0.18 at post-test; d = 0.27 at follow-up) or interaction effects were found
											2. Self-efficacy	Adapted from previous studies [52–55]; 5 items	Significant increase in both groups at post-test (d = 1.27 for INT; d = 1.34 for COM) and maintained at follow-up (d = 1.22 for INT; d = 1.48 for COM); no group (d = 0.16 at post-test; d = 0.07 at follow-up) or interaction effects were found
											3. Gatekeeper skills	Adapted from Observational Rating Scale of Gatekeeper Skills (ORS-GS) Scoring System [54, 55]; 5 items	Higher score in INT compared to COM at post-test (d = 0.46); no group difference at follow-up (d = 0.25)
											4. Gatekeeper behavior	Self-reported referrals: Self-developed items; 1 item	No difference between INT and COM at follow-up (d = 0.01)
Klingman [40]	Northern Israel	30	Teachers and counselors	NR	0%		Gatekeeper training in group-oriented workshop format	Gatekeeper training in problem-oriented workshop format	3 h; NR	NA	1. Knowledge	General knowledge: Self-developed items, 13 items	Both groups scored significantly higher at post-test (d = 3.30 for INT; d = 3.63 for COM); no significant difference between groups (d = 0.00)
												Identification of warning signs: self-developed items, 12 items	Both groups scored significantly higher at post-test (d = 1.36 for INT; d = 1.53 for COM); no significant difference between groups (d = − 0.23)
												Knowledge about prevention: Self-developed items, 7 items	Both groups scored significantly higher at post-test (d = 1.59 for INT; d = 0.68 for COM); problem-oriented group showed significantly more knowledge than group oriented group (d = 0.68)
											2. Self-efficacy	Personal competence: Self-developed items, 7 items	Both groups scored significantly higher at post-test (d = 1.04 for INT; d = 1.24 for COM); no significant difference between groups (d = − 0.15)
Tompkins et al. [21]	The pacific Northwest	INT = 106 CON = 35	School personnel	NR	22.6%	QPR	Gatekeeper training	No intervention	1 h; 27.7% %	3 months, 72.3%	1. Knowledge	Knowledge of QPR: Adapted from previous studies; 15 items	Significant increase in INT compared to COM in at post-test (d = 1.52) but not maintained at follow-up (d = 0.46)
												Self evaluation of knowledge: Adapted from previous studies; 6 items	Significant increase in INT compared to COM in at post-test (d = 1.63) but not maintained at follow-up (d = 0.76)
											2. Attitudes	Adapted from previous studies; 3 items	Significant increase in INT compared to COM in 1 of the 3 items at post-test (d = 0.93) and follow-up (d = 0.24)
											3. Likelihood to intervene	Likelihood to question about suicide intent: Adapted from previous studies; 4 items	Significant increase in INT compared to COM at post-test (d = 1.51) and follow-up (d = 1.26)
												Likelihood to intervene: Adapted from previous studies; 7 items	Significant increase in INT compared to COM at post-test (d = 0.47) and follow-up (d = 0.33)
											4. Self-efficacy	Adapted from previous studies; 3 items	Significant increase in INT compared to COM at post-test (d = 0.75) and follow-up (d = 0.51)
Wyman et al. [29]	United States	INT = 166 CON = 176	School staff	44.5 (range = 22–75)	18.1%	QPR	Gatekeeper training	Waitlist control	1.5 h; NA	1 year; 22.6%	1. Knowledge	QPR knowledge: Self-developed items; 14 items	Significant intervention effect at follow-up (d = 0.44)
												Self-evaluation knowledge: Self-developed items; 9 items	Significant intervention effect at follow-up (d = 0.74)
											2. Self-efficacy	Self-developed items; 7 items	Significant intervention effect at follow-up (d = 0.95)
											3. Gatekeeper behavior	Asking students about suicide: Self-developed items; 1 item	No intervention effect at follow-up (d = 0.11); significant intervention by baseline interaction effect at follow-up
												Referral behaviors: Self-developed items; 6 items	No intervention effect at follow-up (d = 0.09)
*Controlled trials without a pre-test*													
Angerstein et al. [49]	North Texas, United States	INT = 53 COM1 = 26 COM2 = 46	Counselors (N = 79) and building administrators (N = 71)	NR	NR	Project SOAR	Gatekeeper training	No intervention	18 h; 12.8%	NA	1. Knowledge	Suicide awareness Survey; Self-developed items; 10 items	Significant higher score in INT compared to COM1 at post-test (d = 2.04); significant higher score in INT compared to COM2 at post-test (d = 1.12)
											2. Attitudes	Suicide awareness Survey; Self-developed items; 5 items	Significant higher score in INT compared to COM1 at post-test (d = 0.83); no significant difference between INT and COM2 at post-test (d = 0.32)
Reis and Cornell [41]	Virginia, United States	INT = 238 CON = 172	Counselors (N = 147) and teachers (N = 263)	NR	NR	QPR	Gatekeeper training	No intervention	1–3 h; NA	4.7 months (range from 1–22 months)	1. Knowledge	The Student Suicide Prevention Survey; Self-developed items; 7 items	Significant intervention effect at follow-up (d = 0.20)
											2. Gatekeeper behavior	The Student Suicide Prevention Survey; Self-developed items; 3 items	INT made more contract with students (d = 0.44), but made fewer referrals for mental health services (d = 0.37) and questioned fewer potentially suicidal students (d = 0.36) than did COM
*Before- and after comparison*													
Angerstein et al. [49]	North Texas, United States	62	Counselors	NR	NR	Project SOAR	Gatekeeper training	NA	8 h; 28%	NA	1. Knowledge	Adapted from previous study [56]; 16 items	Significant increase in knowledge at post-test for high school of both groups (d for group A = 1.75; d for group B = 0.84) and for middle school of group B (d = 1.48) but not for group A (d = 0.24)
Mackesy-Amiti et al. [46]	United States	205	School personnel and community representatives	NR	28.3%	Preparing for Crisis	Gatekeeper training	NA	4 h; NR	NA	1. Knowledge	PFC Knowledge test; Self-developed items; 25 items	Significant increase in knowledge at post-test (d = 0.79)
Robinson et al. [47]	Australia	213	School welfare staff	42.5 (SD = 10.6)	14.1%		Gatekeeper training	NA	1 or 2 days; 13.2%	6 months; 20.1%	1. Knowledge	Knowledge of Deliberate Self-harm Questionnaire [57]; 10 items	Significant increase in knowledge at post-test (d = 0.56). 26% of participants who rated at high level at post-test demonstrated a reduction in knowledge; while 70% of those who had moderate level at post-test demonstrated increase in knowledge at follow-up
											2. Attitudes	Attitudes towards Children who Self-Harm Questionnaire; [57]; 17 items	No significant change was observed at post-test (d = − 0.05) and follow-up (d = 0.08)
											3. Gatekeeper skills	(1) Skills in dealing with mental illness: Self-developed item; 1 item (2) Skills in dealing with self-harm: Self-developed item; 1 item	Significant increase in perceived skills at post-test (d = 0.78) and maintained at follow-up (d = − 0.66) Significant increase in perceived skills at post-test (d = 1.40) and maintained at follow-up (d = − 0.20)
											4. Self-efficacy	(1) Confidence in dealing with mental illness: Self-developed item; 1 item (2) Confidence in dealing with self-harm: Self-developed item; 1 item	Significant increase in confidence at post-test (d = 0.58) and maintained at follow-up (d = − 0.14) Significant increase in confidence at post-test (d = 1.12) and maintained at follow-up (d = − 0.09)
Suldo et al. [43]	United States	121	School Psychologists	41.1 (SD = 10.8)	18.3%		Gatekeeper	NA	4 h; 53%	9 months; 66.1%	1. Knowledge	Knowledge on prevention, intervention, postvention, and overall knowledge score: Adapted from previous study [58]; 15 items	Significant time effect in all 4 scores at post-test (d = 0.45, 0.37, 0.75 and 0.80, respectively). Significant decrease in knowledge on prevention (d = − 0.69), postvention (d = − 0.52), and overall knowledge score (d = − 0.46) from post-test to follow-up. Score on intervention maintained from post-test to follow-up (d = 0.15)
											2. Self-efficacy	Perceived competence in suicide-related professional activities of prevention, assessment, referral, counselling and postvention: Adapted from previous study [58]; 5 items	Significant increase in confidence to execute all 5 suicide-related professional activities at post-test (d = 0.72, 0.62, 0.60, 0.30, and 0.61, respectively), the effect was maintained in all of the activities at follow-up (d = − 0.36, − 0.03, − 0.04, − 0.02 and − 0.17, respectively)
												Confidence in working with diverse youth, in terms of culture, English language speaking, disability, sexual orientation and strong religious affiliation) around suicide issues: self-developed items: 5 items	Significant increase in all 5 populations at post-test (d = 0.58, 0.70, 0.59, 0.64 and 0.51); the effect was maintained among the first four types of diverse youths (d = 0, − 0.07, − 0.16, 0.12, respectively), and further increase in youth with strong religious affiliations (d = 0.22) at follow-up
Walsh et al. [22]	United States	220	School personnel	NR	23%		Gatekeeper training	NA	1.5 h; 18.1%	NA	1. Likelihood to intervene	Adapted from previous studies [59, 60]; 1 item	Significant increase in likelihood to intervene at post-test (d = 0.69)
											2. Self-efficacy	Confidence: Adapted from previous studies [59, 60]; 1 item	Significant increase in confidence at post-test (d = 0.59)
												Comfort in asking: Adapted from previous studies [59, 60]; 1 item	Significant increase comfort in asking at post-test (d = 0.68)
Johnson et al. [42]	Midwest, United States	36	High school and middle school staff	NA	NA	QPR suicide prevention program	in-person QPR Gatekeeper training + online conference work group	NA	three 90 min sessions; 100%	Monthly email for a 3 month time period following training; 100%	1. Knowledge	QPR Knowledge: self-developed survey; 9 items	Significant increases in means of all knowledge items at post-test (d ranged from 1.11 to 1.90)
Lamis et al. [44]	Atlanta, Georgia, United States	700	School teachers (N = 620); school administrators (N = 35); classroom aids (N = 26); guidance counselors (N = 19)	40.24 (SD = 12.03)	20.4	Act on FACTS: Making Educators Partners in Youth Suicide Prevention (MEP)	Online gatekeeper training	NA	2 h; 100%	NA	1. Knowledge	Suicide knowledge: self-developed items; 15 items	Significant increase in knowledge at post-test (d = 1.51)
											2. Self-efficacy	Self-developed items; 7 items	Significant increase in self-efficacy at post-test (d = 1.66)
Santos et al. [45]	Coimbra, Portugal	66	School primary healthcare professionals	41.5 (MIN = 26, MAX = 61)	7.6	“+ Contigo” training	Gatekeeper training	NA	three 21 h courses; 100%	NA	1. Knowledge	Knowledge about suicide prevention: Adapted from Suicide Behavior Attitude Questionnaire [61]; 13 items	Significant increase in knowledge at post-test^a^
											2. Attitudes	Adapted from Suicide Behavior Attitude Questionnaire [61]^a^: 1) negative feelings towards individuals with suicidal behaviors; item no. NA 2) attitudes towards the right to suicide; item no. NA	No significant differences in attitudes toward individuals with suicidal behaviors or towards the right to suicide at post-test^a^
											3. Gatekeeper skills	Perceived professional skills: Adapted from Suicide Behavior Attitude Questionnaire [61]; item no. NA	Significant increase in perceived skills at post-test^a^
Groschwitz et al. [48]	Baden-Wuerttemberg, Germany	236	school psychologists (N = 22), school social workers (N = 143), teachers (N = 55) and other school staff (N = 15)	NA	16.9	Strong Schools against Suicidality and Self-Injury (4S) program	Workshops	NA	2 days; 99.6%	6 months; 20.8%	1. Knowledge	Adapted from Mental Health First Aid Training [62] and the Teacher Knowledge and Attitudes About Self-Injuries Questionnaire [63]; 8 items	Significant increase in perceived knowledge at post-test (d = 1.67) and maintained at follow-up (d = 1.41)
											2. Self-efficacy	Confidence in Gatekeeper skills: Adapted from Mental Health First Aid Training [62] and the Teacher Knowledge and Attitudes About Self-Injuries Questionnaire [63]; 8 items	Significant increase in confidence at post-test (d = 1.68) and maintained at follow-up (d = 1.56)
											3. Attitudes	Adapted from Attitudes towards Children Who Self-harm Questionaire [57]; 7 items	No significant differences in attitudes toward suicidality at post-test (d = 0.44) or at follow-up (d = 0.23)

*NA* relevant information was not available

^a^The effect size was not presented due to the necessary information not available

### Study characteristics

The characteristics of the included studies are presented in Table 1. Fifteen programmes were described in the 14 included studies. Approximately 3050 gatekeeper participants were covered in these programmes, only one of which solely involved female participants [40]. Participants included teachers, counsellors, social workers, and psychologists. Nine studies were conducted in the United States.

In terms of intervention, five out of the ten included studies used the QPR approach. Certified trainers led a single-session training which commonly lasted for 1–3 h [13, 21, 29, 41], whereas one study performed three 90 min sessions [42]. Three of these studies reinforced the intervention following the standard QPR programme. Wyman et al. [29] conducted a 30 min QPR refresher after several months. Cross et al. [13] provided an additional 25 min role play practice right after the QPR training to the intervention group. Johnson et al. [42] further created an online conference work group. Five other studies performed diverse interactive trainings [22, 40, 43–45]. Mackesy-Amiti et al. [46] conducted a 4 h postvention programme which prepared participants for developing and implementing a crisis plan for sudden loss as a way for suicide prevention. Two other 2 day programmes [47, 48] focused on the management of self-harm, a high risk factor of suicide. Angerstein et al. [49] formally evaluated a comprehensive school-based suicide programme, the Project SOAR, among two different samples.

In terms of study design, six studies had a follow-up evaluation and the duration of follow-up ranged from 3 to 22 months. A comparison group was used in six studies, though only two studies employed a random assignment of participants [13, 29], and only one study employed intent-to-treat analyses [29]. None of the included studies concealed allocation, or kept deliverers blind during the interventions (Table 2). Four studies compared the effect of gatekeeper training with a control group which received no intervention or waitlist intervention [21, 29, 41, 49]. One study compared the efficacy of QPR plus behavioural activation over QPR [13] and another study compared the efficacy of gatekeeper training delivered in a group format over a problem-oriented format [40]. In terms of measures, half of the studies reported a wide variation in the reliability of measure items across studies and constructs.

**Table 2:** Methodological quality of the controlled trials included in the systematic review (N = 6)

Study	Random assignment	Allocation concealment	Blind	Intention-to-treat analysis
Angerstein et al. [49]	No	No	No	No
Cross et al. [13]	Yes	No	No	No
Klingman [40]	No	No	No	No
Reis and Cornell [41]	No	No	No	No
Tompkins et al. [21]	No	No	No	No
Wyman et al. [29]	Yes	No	No	Yes

### Effectiveness of school-based gatekeeper training for adolescent suicide prevention

#### Knowledge

Thirteen studies assessed the outcome of gatekeepers’ knowledge; all of which showed benefits in increasing knowledge. Seven of these studies employed or adapted measure items from previous studies [13, 21, 43, 45, 47–49]. Of the four studies with a pretest–posttest-control (PPC) design, both of the two trials which compared QPR with a blank control reported significant training condition effects on improving declarative knowledge and self-perceived general knowledge [21, 29]. The other two trials testing different types of gatekeeper training yielded mixed results; the superiority of an additional rehearsal to standard QPR was not found [13], while the gatekeeper training in a problem-oriented format was significantly better than a group-oriented format in increasing the knowledge about prevention, but not the general knowledge or knowledge in identification of warning signs [40]. Despite significant increases in knowledge at immediate post-test found for all gatekeeper training conditions in these four studies, one study further showed that such a positive effect was not maintained at a 3 month follow-up [21]. Both of the studies with a posttest only with control (POC) design compared the gatekeeper raining with a null control and found significant higher scores on factual knowledge about suicide in the intervention group [41, 49].

All the eight studies with a single-group pre-post-test (SGPP) design detected a significant increase in specific knowledge outcomes immediately after the gatekeeper trainings, including knowledge about suicide prevention [42, 44, 45, 49]. suicidality-related self-injury [47, 48], crisis preparing for suicide postvention [46], and comprehensive suicide-related practices [43]. However, findings on the long-term effects of gatekeeper trainings were inconsistent. Groschwitz et al. [48] observed the maintenance of the significant gain in knowledge about suicidality and self-injury at the 6 month follow-up. Robinsons et al. [47] reported a reduction in knowledge at the 6 month follow-up among participants rated at the high knowledge level at post-test, whilst a steady increase among those at the moderate level. Suldo et al. [43] found that only score on knowledge about intervention was maintained at 9 month follow-up, whereas scores on that about prevention, postvention and total knowledge decreased significantly from post-test to follow-up.

Moderators were also identified for the above gatekeeper training effects. Individuals with a lower knowledge level prior to the trainings evidenced greater gains [21, 29, 47]. Tompkins et al. [21] showed a significant improvement in QPR knowledge among teachers and administrators but not support staff. Angerstein et al. [49] detected a notable knowledge increase in those trained at both target high schools but at only one of the two middle schools.

#### Gatekeeper skills

Three studies assessed the outcome of gatekeeper skills and all of them showed significant positive effect. Cross et al. [13] showed that participants in the QPR plus behavioral rehearsal condition demonstrated significantly higher total gatekeeper skills than those in the QPR condition, but the 3 month follow-up scores significantly decreased. Specifically, the effect was found on general communication but not on suicide-related skills. Robinson et al. [47] reported a positive change in the skills of dealing with self-harm at post-test, which was maintained at the 6 month follow-up. The most improvement occurred among those who reported low and moderate level of skills prior to the course. Finally, Santos et al. [45] also found a significantly higher level of perceived professional skills right after the gatekeeper training.

#### Attitude towards adolescent suicide

Five studies measured the change in attitude towards adolescent suicide. A positive effect of gatekeeper trainings was observed in two controlled trials; one found a higher score on attitudes about suicide in the training group compared to one of the control groups [49]; while the other observed a significant increase only in one (“suicide is preventable”) of the three attitudes items at post-test and 3 month follow-up [21]. None of the three studies with a SGPP design showed a significant time effect of gatekeeper trainings on the attitudes towards suicidal (or related) behaviors and suicide prevention [45, 47, 48]. The last four studies employed or adapted the items from previous studies.

#### Self-efficacy

All nine studies that assessed change in self-efficacy reported positive effects. Five had adapted scales from previous studies [13, 21, 22, 48]. The four studies with a PPC design reported a significant increase in self-efficacy for identifying and responding to suicidal individuals after training and/or at a long-term follow-up. The intervention was also found to be more effective than the blank control group [21, 29]. However, comparison between different types of gatekeeper training indicated no significant condition effect [13, 40].

The five studies with a SGPP design documented a significant increase in trainees’ confidence in dealing with suicidality immediately at post-training. Long-term effects were inconsistent in three studies that assessed them. Two showed that gains in self-efficacy were maintained at 6 month follow-up [47, 48]. The third, Suldo et al. [43] reported a steady increase from post-test to 9 month follow-up in participants’ confidence in their abilities to execute the suicide-related professional activities; and in the confidence of working with youth with strong religious affiliations but not with those from diverse cultures, with disabilities, with diverse sexual orientations or those who were English language learners.

Participants’ profession roles and professional experience were identified as potential moderators of the gatekeeper training effects. Lamis et al. [44] revealed a significantly larger increase in self-efficacy at post-test among teachers and classroom aids than among guidance counsellors and school administrators. Groschwitz et al. also found teachers improved in confidence most, followed by school social workers and school psychologists [48]. Several studies consistently showed that participants with less knowledge and experience around suicide issues prior to the trainings demonstrated greater gains in self-efficacy [21, 47, 48].

#### Likelihood to intervene

Two studies adapted items from previous research to evaluate the outcome of self-reported likelihood to intervene; both revealed a positive effect. Tompkins et al. [21] reported a significant increase in the likelihood to question a student about suicide intent, as well as the likelihood to intervene in the intervention group compared to the null-control group at post-test and 3 month follow-up. Individuals with prior suicide prevention training evidenced more pre-post changes in the likelihood to question suicide intent. Walsh et al. [22] also detected an increase in the likelihood to directly question a young person about suicide intent from pre-test to post-test.

#### Gatekeeper behaviour

Three controlled trials evaluated the effects on gatekeeper behaviour with self-developed items, and two of them found positive effects on specific behaviours. Wyman et al. [29] found that the gatekeeper training effect on asking students about suicide only presented itself at the 1 year follow-up among staff with such experience at baseline, and no overall effect for suicide identification behaviour was illustrated. Reis and Cornell [41] found that the QPR training group made more contract with students, but unexpectedly, questioned fewer potentially suicidal students and referred fewer students to mental health services than did the null-control group at the 4.7 month follow-up. Cross et al. [13] further showed that an additional behavioural rehearsal to the standard QPR did not significantly increase the number of referrals at the 3 month follow-up.

## Discussion

Given the adverse impact of suicide, there is an urgent need to identify ways to effectively reduce suicide among adolescents. In response to this significant health concern, there has been a surge of programmes using the gatekeeper approach for reducing adolescent suicide. The present study conducted a systematic review of the effectiveness of school-based gatekeeper training for adolescent suicide prevention on gatekeepers’ self-reported knowledge, skills, attitudes, and behaviours relating to the detection of and responses to suicidality. It is important to point out that direct comparisons between studies included in the systematic review are difficult due to the tremendous heterogeneity in sample characteristics, the nature of the comparison groups, mode of intervention, intensity and duration of intervention, outcome measures and length of follow-ups. Nevertheless, findings from the systematic review provide some evidence that gatekeeper training programme for adolescent suicide prevention are generally effective in improving participants’ knowledge and skills, while mixed evidence exist with regards to changing participants’ attitudes and gatekeeper behaviour.

Results from the systematic review show that most of the studies evaluated the effectiveness of the training in improving knowledge as well as self-efficacy, and there is established evidence to support such improvements. Such positive effects were maintained at follow-up. There is also evidence that school-based gatekeeper training is effective in improving participants’ skills and likelihood to intervene, although the number of studies measuring these outcomes are relatively small. Since most of the gatekeeper programmes aim at addressing signs of suicide and improving participants’ skills in intervening with at-risk individuals, it is conceivable that they can be effective in improving participants’ knowledge, self-efficacy and skills. It is further reported that the effect of gatekeeper training is comparable with those with an additional behavioural rehearsal component [13], suggesting that school-based gatekeeper training can potentially be a useful approach in preventing adolescent suicide.

Contrary to our expectation, mixed evidence exists as to the effectiveness of gatekeeper training in changing participants’ attitudes. Results are surprising given that one of the key focuses of gatekeeper training programme is to improve participants’ attitudes. It is, however, important to note that in one study a ceiling effect was seen in half of the items at baseline. This indicated that only limited improvement could be shown on the measures being used [47]. It might therefore be plausible that most of the participants have already shown positive attitudes towards adolescent suicide before receiving gatekeeper training. The heterogeneity in operationalization and measures used for attitudes in various studies might also explain the mixed results. More studies are warranted to investigate the effect of school-based gatekeeper training in improving participants’ attitudes towards adolescent suicide.

Only three of the included studies measured changes in gatekeeper behaviour, and the mixed results found imply that changes in knowledge and skills in suicide prevention may not translate directly to behavioural change. As most of the studies have a relatively short follow-up time, it may not be long enough to capture the change of behaviour among the participants. Unexpectedly, one study found that gatekeeper training resulted in participants in the intervention group questioning and referring a lower number of at-risk students to mental health services. The authors speculated that the gatekeeper training might have improved participants’ confidence and knowledge in adolescent suicide prevention, as well as their ability in assessing students’ abnormal behaviour without the need to ask questions [41]. Establishing contact with at-risk students is the very first step in suicide intervention. It is therefore imperative to examine how the change in knowledge and skills can be translated into change in gatekeeper behaviour so that adolescents who are at risk of suicide could be approached and intervened effectively. Inconsistency in the effectiveness on ultimate gatekeeper behaviour and its correlates could also be explained by a study reporting negative help seeking attitudes among student suicide attempters [29]. The study strongly recommended an integration of the gatekeeper program with interventions on students’ help-seeking behaviour, to help facilitate an open communication [29].

It is important to note that the quality of the studies may have a huge effect on the conclusions that can be drawn. The present review found many of the studies to be of low methodological quality. While the use of RCT is regarded as the best design in delineating cause-and-effect relationships and minimizing confounding variables, the majority of the controlled studies did not use proper randomization and none used allocation concealment when assigning participants. The use of pre- and post- intervention comparisons or non-equivalent control groups was prevalent. No studies kept programme deliverers blinded during the research. Only one study used intent-to-treat analysis to take into account the participants who were lost to follow up. A huge variation was also found on the measures used, with a majority of them reporting the use of self-developed measures. In addition, there is a dearth of studies measuring the effectiveness of school-based gatekeeper programmes in decreasing rates of suicidal ideation, suicide attempts, or deaths by suicide. There is an urgent need to design a high-quality gatekeeper training programme evaluated with psychometrically sound outcome measures.

In addition to the efficacy of gatekeeper approaches, the practical implementation of a specific training program may also greatly affect its effectiveness across different contexts in terms of notable improvements in the target cognitions and behaviours [50]. The assessment of implementation outcomes using high-quality instruments is critical to identifying the most optimal implementation strategies [51] However, only one of the included studies quantitatively measured the acceptability and feasibility of the proposed program [22]. Moreover, developing standardised evaluation methods for implementation science would contribute to the appraisal and comparison of diverse gatekeeper training programs [51].

## Limitations

There are several limitations that should be noted. First, the present review was restricted to English articles; there is a possibility that some articles in other languages may have been overlooked in the review. Second, the literature search was conducted in only four databases. Nevertheless, the databases included were deemed the most relevant ones to adolescent suicide and articles that did not explicitly mention gatekeeper training in their title or abstracts were retained in the first screening, and their full-texts were reviewed before a decision was made. Third, although a positive finding on most outcomes was observed, no conclusion could be made as to the extent of the benefits which were due to social or group effect. Fourth, this study reviews the evidence on changes in gatekeepers’ self-reported cognitive outcomes and behaviour as proxy indicators of reduction in suicide-risk. Few included studies have attempted to relate these changes to those in rates of successful or attempted suicide despite a large number of individual adolescents whose gatekeepers will have received the forms of training and support we have reviewed here. Fifth, the present review did not specifically examine the components which may make the programme effective. Sixth, publication bias might exist in the review, as the present study did not systematically search for articles in the grey literature. Future studies should seek to include other indicators of the effectiveness of school-based gatekeeper training and to conduct a wider review with studies not formally published in the research literature. Lastly, qualitative synthesis of results is inherent to the nature of a systematic review. However, effect size was calculated and presented for each study. Meta-analysis would not be possible on the literature identified for this topic due to the great heterogeneity observed in the study characteristics and limited data on specific outcome measures (e.g. gatekeeper skills).

## Conclusion

The present study conducted a systematic review on the effectiveness of school-based gatekeeper training for adolescent suicide prevention. Findings suggest that school-based gatekeeper training is effective in improving participants’ knowledge, skills, self-efficacy and likelihood to intervene, while mixed evidence exists in changing participants’ attitudes and gatekeeper behaviour. Methodological issues, such as lack of RCT and the inability to use validated measures, jeopardize the conclusions that can be drawn from the studies. More high-quality studies with longer follow-up periods are warranted to ascertain the effect of school-based gatekeeper training in improving participants’ knowledge, skills, attitudes towards adolescent suicide and gatekeeper behaviour. Such studies should also seek to include long term outcomes such as suicide attempts or behaviour.

## Relevance for clinical practice

Findings of the present study have important implications for the design of adolescent suicide prevention programmes. Findings suggest that a school-based gatekeeper approach, training teachers or school staff to identify and intervene on behalf of at-risk students, could be implemented in programmes aimed at adolescent suicide prevention. Teachers and school staff can play an important role and school potentially serves as a useful setting in which such programmes could be implemented. Mental health professionals should collaborate with schools in the design and implementation of further research to adequately evaluate and establish the benefits of such adolescent suicide prevention approaches.
